# Systemic Infusion of Expanded CD133^+^ Cells and Expanded CD133^+^ Cell-Derived EVs for the Treatment of Ischemic Cardiomyopathy in a Rat Model of AMI

**DOI:** 10.1155/2019/4802578

**Published:** 2019-12-01

**Authors:** Addeli B. B. Angulski, Luiz Guilherme A. Capriglione, Fabiane Barchiki, Paulo Brofman, Marco A. Stimamiglio, Alexandra C. Senegaglia, Alejandro Correa

**Affiliations:** ^1^Carlos Chagas Institute, Oswaldo Cruz Foundation (FIOCRUZ), Curitiba 81350-010, Brazil; ^2^Core for Cell Technology-School of Medicine, Pontifical Catholic University of Paraná, Curitiba 80215-901, Brazil

## Abstract

Myocardial infarction is a leading cause of death among all cardiovascular diseases. Cell therapies using a cell population enriched with endothelial progenitor cells (EPCs), expanded CD133^+^ cells, have promise as a therapeutic option for the treatment of ischemic areas after infarction. Recently, secreted membrane vesicles, including exosomes and microvesicles, have been recognized as new therapeutic candidates with important roles in intercellular and tissue communication. Expanded CD133^+^ cells have the ability to produce extracellular vesicles (EVs); however, their effect in the context of the heart is unknown. In the present study, we evaluated the effectiveness of the systemic application of expanded CD133^+^ cells and expanded CD133^+^ cell-derived EVs for the treatment of ischemic cardiomyopathy in a rat model of acute myocardial infarction (AMI) and examined the hypothesis that the EVs, because of their critical role in transferring regenerative signals from stem cells to the injured tissues, might elicit an equal or better therapeutic response than the expanded CD133^+^ cells. We demonstrate that the systemic application of expanded CD133^+^ cells and EVs has similar effects in infarcted rats. Few animals per group showed improvements in several heart and kidney parameters analyzed, but not significant differences were observed when comparing the groups. The systemic route may not be effective to treat ischemic cardiomyopathy; nonetheless, it may be a beneficial therapy to treat the side effects of AMI such as kidney damage.

## 1. Introduction

Cardiovascular diseases (CVD) contribute to approximately 30% of global morbidity and mortality, therefore representing a major public health concern [1]. Among the several types of CVD, acute myocardial infarction (AMI) remains a major worldwide medical problem that results from coronary artery occlusion and subsequent hypoxic ischemic injury [2]. Several studies have shown that heart failure may induce acute or chronic kidney injury and, conversely, that kidney disease itself might be a contributor to severe cardiac damage. Thus, a derangement of cardiac function can lead to renal disease, which is referred to as cardiorenal syndrome [3]. The demonstration that the infusion of bone marrow-derived stem cells in the contracting wall of the infarcted zone in mice can restore myocardial damage and improve heart function has emerged as a promising therapeutic strategy for myocardial regeneration and the restoration of ventricular contractility [4]. A population of cells expressing the CD133 marker and enriched with endothelial progenitor cells (EPCs) has been considered highly potent cells capable of recovering injured tissues, including the postischemic myocardium [5, 6]. In the past few years, CD133^+^ cells have been evaluated in clinical studies aiming to treat patients with myocardial infarction, therefore opening new avenues for the treatment of ischemic areas [5]. Within this context, our group recently reported that transplanted expanded CD133^+^ cells ameliorated the infarcted heart and were suitable for the regeneration of the vascular system in a preclinical study, demonstrating strong potential for vascular regeneration [7]. Despite the demonstrated capacity of CD133^+^ cells to integrate into ischemic tissues and contribute to healing by promoting local angiogenesis [5, 6, 8], some studies have also suggested that the beneficial effects exerted by these cells are more likely indirect and dependent on their paracrine activities, including the secretion of extracellular vesicles (EVs) [9, 10]. These natural nanoscale lipid bilayer vesicles are effective mediators of cell-to-cell communication, at least partially by transferring distinctive molecules of proteins, mRNAs, microRNAs, and other noncoding RNAs specific to the parent cell type [11–13]. EVs include, among others, exosomes and microvesicles (MVs). Exosomes are released via exocytosis from multivesicular bodies of the late endosome and typically have diameters ranging from 30 to 100 nm. MVs directly bud from the plasma membrane and exhibit a diameter ranging from 100 nm to 1 *μ*m. Given the multiplicity of signals carried by these vesicles through the horizontal transfer of functional molecules, CD133^+^-secreted EVs have been tested as a free cell therapy in animal models and have been shown to protect/repair injured tissues [14, 15]. However, to date, none of these studies have been carried out using EVs isolated from expanded CD133^+^ cells derived from human umbilical cord blood in an animal model of cardiac ischemia. In the present study, we evaluated the effectiveness of the systemic application of expanded CD133^+^ cells and expanded CD133^+^ cell-derived EVs for the treatment of ischemic cardiomyopathy in a rat model of AMI. We examined the hypothesis that the EVs, because of their critical role in transferring regenerative signals from stem cells to the injured tissues, might elicit an equal or better therapeutic response than the expanded CD133^+^ cells.

## 2. Materials and Methods

### 2.1. Ethics

Human umbilical cords were collected at Maternidade Victor Ferreira do Amaral (Curitiba, Paraná, Brazil). Samples were obtained after signed informed consent was provided by the patients' legal representatives. This study was reviewed and approved by the Research Ethics Committee and the Ethics Committee on the Use of Animals of Pontifícia Universidade Católica do Paraná (approval numbers 1366 and 763, respectively).

A blind and completely randomized study was conducted comparing four groups divided as follows: control group (control): no surgical procedure or treatment was performed and its purpose was to establish the parameters evaluated in healthy animals (*n* = 6); AMI group (vehicle): rats were submitted to acute myocardial infarction and treated with PBS (*n* = 8); AMI group (EVs): rats underwent acute myocardial infarction and were treated with CD133^+^ cell-derived LVs (*n* = 8); and AMI group (CD133^+^): rats underwent acute myocardial infarction and were treated with cells (*n* = 8).

### 2.2. CD133^+^ Cell Isolation, Expansion, and Characterization

CD133^+^ cells were isolated and expanded as previously described by our group [16]. Briefly, the isolation of mononuclear cells (MNCs) was performed according to the method of Boyum [17], modified using a Histopaque™ 1.077 density gradient (Sigma-Aldrich, São Paulo, Brazil). EPCs (CD133^+^) were selected using CD133-coupled magnetic microbeads (Miltenyi Biotec) according to the manufacturer's instructions. CD133^+^ cells were plated at a density of 1 × 10^5^ cells per cm^2^ in culture flasks and grown in supplemented EBM-2 (Endothelial Cell Growth Medium) (Lonza Clonetics). The vials were incubated in a humidified incubator at 37°C with 5% CO_2_ tension. The culture medium was changed every 3-4 days until the cells reached confluence. When confluent, the adherent cells were dissociated using 0.25% trypsin-EDTA and replated at a concentration of 1.3 × 10^4^ cells per cm^2^. For the experiments, cells were used between passages 6 and 8. The phenotypic characterization of CD133^+^ cells was performed according to the methodology previously reported by our group (Correa et al., 2018). To evaluate the viability of CD133^+^ cells at the time of EV isolation, apoptosis and necrosis assays were performed as previously described by our group [18].

### 2.3. Isolation of Extracellular Vesicles

EVs derived from expanded CD133^+^ cells were isolated from the conditioned medium obtained from CD133^+^ cell culture. After the cells reached confluence, they were washed twice with EBM-2 (Lonza Clonetics) supplemented with 2% FBS depleted of vesicles to avoid crosscontamination. After 24 h of incubation, the conditioned medium was centrifuged at 700 × *g* for 5 min to remove cellular debris and then centrifuged at 4000 × *g* for 20 min to remove apoptotic bodies. For EV collection, the supernatant of the cell culture was ultracentrifuged at 100,000 × *g* for 1 h and 20 min at 4°C and discarded, and the vesicles were then recovered from the bottom of the tubes. This procedure was performed for three consecutive days, and at the end of the third day, the vesicles were diluted with 30 mL of PBS and subjected to a second ultracentrifugation at 100,000 × *g* for 2 h at 4°C for cleaning. Finally, the purified EVs from CD133^+^ cell culture were resuspended in small volumes of PBS, after which the protein concentration of EV samples was determined using a Qubit® 2.0 (Life Technologies™, Invitrogen) assay. All fractions were stored at -80°C until use.

### 2.4. Transmission Electron Microscopy

Optimal concentrations of purified EVs derived from expanded CD133^+^ cells were loaded onto 300-mesh niquel/formvar-coated grids (Electron Microscopy Science), and after 1 h of absorption to the formvar, the grids were fixed with 4% paraformaldehyde (Electron Microscopy Science) for 10 min. For immunogold staining, the grids were previously immersed in blocking buffer (PBS/2% BSA) for a block/permeabilization step for 30 min before labeling with the primary antibodies at the appropriate dilution for 1 h at room temperature (1 : 200 anti-CD63, ab59479, Abcam; 1 : 100 anti-CD105, 555.690, BD Bioscience; and 1 : 100 anti-CD31, 555.444, BD Bioscience). After rinsing, the grids were incubated with a specific antibody conjugated to 15 nm gold nanoparticles (1 : 20 25.233, Electron Microscopy Science) for 40 min at room temperature. The grids were postfixed in 2.5% glutaraldehyde (Sigma-Aldrich) for 10 min and then stained for contrast using 2% uranyl acetate for 10 min. The samples were examined on a transmission JEOL JEM-1011 electron microscope (JEOL Ltd.) at the Electron Microscopy Facility (Universidade Federal de Santa Catarina (UFSC) and Universidade Federal do Paraná (UFPR)) operating at an acceleration voltage of 80 kV.

### 2.5. Nanoparticle Tracking Analysis

Analyses of the size distribution and number of EVs derived from expanded CD133^+^ cells were performed by the same operator on a NanoSight LM10 (NanoSight Ltd.) instrument. Expanded CD133^+^ cell-derived EVs were mixed by vortexing and subsequently diluted in particle-free PBS (0.02 *μ*m filtered) to obtain a concentration within the recommended measurement range (1‐10 × 10^8^ particles/mL), corresponding to dilutions from 1 : 50 to 1 : 200 depending on the initial sample concentration. Through nanoparticle tracking analysis (NTA), the particles were automatically tracked and sized based on their Brownian motion and diffusion coefficient. Experimental videos were analyzed using NTA 3.6 analytic software after capture in script control mode (3 videos of 60 s per measurement). The results obtained are displayed in a histogram graph as the frequency of size distribution.

### 2.6. Experimental Model of AMI in Rats

This animal model was established according to the methodology previously described by our group [19]. Briefly, for anesthetization, the rats were premedicated prior to anesthetic induction with 2 mg/kg/IM nalbuphine followed by induction (~4%) and anesthetic maintenance (Chiarorn) with isoflurane (~2.5%). Perineural anesthesia of the 3^rd^, 4^th^, and 5^th^ left intercostal nerves with 0.75% lidocaine at a dose of 3 mg/kg was also performed prior to thoracotomy. The surgical technique was performed aseptically. A thoracotomy of the fourth left intercostal space was performed, and then, the left coronary artery was identified and occluded with 6.0 silk thread, approximately 2-3 mm from its origin, between the atrium of the left atrium and the sulcus of the pulmonary artery. In the postoperative period, all the animals received nalbuphine (1 mg/kg/IM, TID for 24 h), meloxicam (5 mg/kg/SC, BID for 24 h), and enrofloxacin (10 mg/kg, IM).

### 2.7. Echocardiographic and Electrocardiographic Evaluations

Transthoracic echocardiography (TTE) was performed 24 h after AMI (pretreatment) and 28 days after expanded CD133^+^ cell or expanded CD133^+^ cell-derived EV transplantation (posttreatment) by an experienced professional who did not have knowledge of the experimental groups. The left ventricle echocardiographic variables were end-diastolic volume (EDV), end-systolic volume (ESV), and left ventricular ejection fraction (LVEF) and were measured using Simpson's method [20]. Rats with an LVEF less than 45%, exhibiting ventricular dysfunction, were included in this study. At 24 h after surgery, electrocardiography was performed in leads DI, DII, DIII, AVR, AVL, and AVF to confirm and evaluate infarction size by determining the electrical axis and pathological Q wave in DI derivation according to Pimentel et al. [21].

### 2.8. Cell and EV Transplantation

The animals were treated 24 h after AMI (soon after the determination of all baseline echocardiographic parameters). Cell or EV transplantation or phosphate-buffered saline (PBS) administration was performed by a single injection in the lateral tail vein under isoflurane anesthesia (induction 4% and maintenance 1.5-2%). The cell or EV transplantation was performed using a 1 mL insulin syringe containing 2 × 10^6^ cells or 50 *μ*g (corresponding to 7.8 × 10^9^ to 14 × 10^9^ EVs/animal) of expanded CD133^+^ cell-derived EVs diluted in 0.6 mL of particle-free PBS (0.02 *μ*m filtered). In the vehicle group, the same syringe was filled with 0.6 mL of PBS and used. After recovery from anesthesia, the rats were returned to their home cages, where they were kept for 28 days under the same conditions described in Section 2.6. After 28 days, the second ECO was performed followed by euthanasia. The rats were humanely euthanized by an anesthetic overdose. Initially, ketamine (100 mg/kg) associated with xylazine (10 mg/kg) was administered by intramuscular injection. After sedation, the animals were placed in a glass induction chamber filled with isoflurane that was used to induce anesthesia. After the confirmation of death, each rat was necropsied.

### 2.9. Histopathology of the Hearts and Kidneys

After euthanasia, the rats were necropsied, and the heart and kidneys were harvested for histopathological analysis. The hearts and kidneys were fixed in paraformaldehyde using the whole animal perfusion fixation technique. Briefly, a preperfusion with PBS was performed by passing a perfusion needle through the incision in the left ventricle until it reached the ascending aorta. The PBS was used to flush the body until the fluid exiting the rat was clear of blood. Thereafter, 4% paraformaldehyde was used as a fixative solution to perfuse the whole body. Perfusion was complete when all muscle contractions had stopped, the liver and mesenteric vessels were blanched, and the desired amount of preservative had passed through the circulatory system. Hearts and kidneys were washed with saline and embedded in a fixative solution of 10% formalin overnight. The formalin-maintained samples were washed in water, dehydrated using an ascending alcohol series, and then embedded in paraffin. Hearts and kidneys were sectioned and stained with Masson trichrome to detect collagen deposition within the myocardium or HE to reveal general histopathology. To analyze the estimated infarct size, slides were examined using ZEN 2.1 lite software (Zeiss). For renal injury evaluation, slices were scanned in an Axio Scan Z1 (Zeiss) with a 20x objective and then analyzed using Zen 2.1 Lite software with a 3% increase. Three central areas were randomly selected in the left and right kidney cortex. Renal damage was evaluated using the scoring system (scale 0 to 4) according to Jablonski et al. [22] for the identification of necrosis in glomeruli and tubules. On this scale, 0 represents no histological damage and 4 represents severe acute tubular necrosis. Scoring was performed in both the preserved and damaged parts of the renal cortex.

### 2.10. Statistical Analysis

The statistical analysis of multiple group comparison was performed using one-way analysis of variance (ANOVA) followed by Bonferroni's test. For the variables that did present a normal distribution (ESV and EDV), Student's *t*-test for paired samples was used. For the variables that did not indicate distribution of normality (LVEF and infarct size), the nonparametric Wilcoxon test was used to compare the medians of each group pre- and posttreatment. Exact Fisher's test for proportions was used to determine whether the rates of occurrence of renal tubular epithelial lesion in the groups were different from each other. Animals that died during the study were excluded from the statistical analysis. *P* values < 0.05 were considered to be statistically significant.

## 3. Results

### 3.1. Acute Myocardial Infarction in Rats

**Figure 1:**
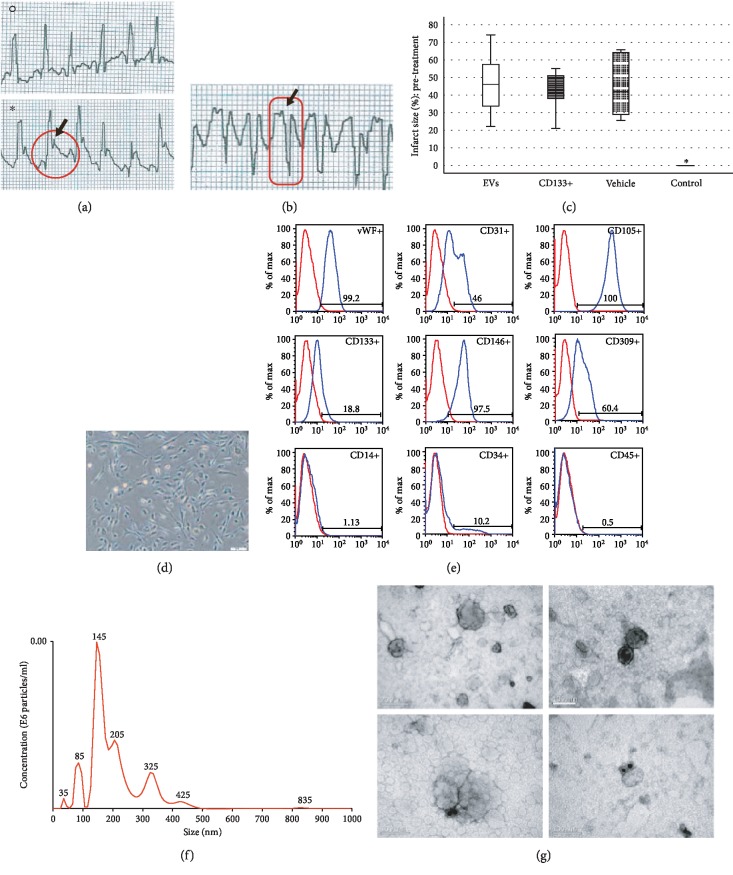
Characterization of the myocardial infarction model, expanded CD133^+^ cells, and expanded CD133^+^ cell-derived EVs. (a) Electrocardiographic tracing of a rat before surgery (°) and after 5 minutes of coronary occlusion (∗). An increase in the T wave and the ST segment (red circle) is observed, which indicates myocardial hypoxia and confirms the success of the procedure. (b) Electrocardiographic tracing in DI lead performed 24 hours after coronary occlusion. The identification of negative Q wave (highlighted in red) demonstrates that the induction of AMI was successful. (c) Echocardiographic evaluations revealed infarct size similarity in all animals before treatment, indicating no significant difference in initial ischemic injury among infarcted groups. The control group showed intact hearts with no evidences of IAM. (d) Representative field showing the endothelial-like morphology of expanded CD133^+^ cells at passage 4 (magnification 200x, scale bars 100 *μ*m). (e) Representative flow cytometry analysis of cell surface markers of CD133^+^ cells at passage 3. The isotype control is shown as a red line histogram. (f) Representative tracing of CD133^+^-EV concentration and size by nanoparticle tracking analysis. (g) Representative transmission electron microscopy (TEM) images of CD133^+^-EVs. The lower panel represents immunogold staining of surface proteins CD31 (left) and CD63 (right).

An acute myocardial infarction (AMI) in rats is commonly used to mimic human ventricular dysfunction leading to heart failure. In this study, we used a rat model of myocardial infarction in which the left anterior descending coronary artery was occluded (Figure 1(a)). The induction of AMI was successful in all 28 rats, leading to a substantial infarcted zone visually observed in the left ventricle immediately after the left anterior coronary artery was occluded. Echocardiography recordings taken 24 h after AMI showed electrical axis deviation to the right and negative Q wave derivation D1 (Figure 1(b)). Data from ECG also confirmed the presence of myocardial infarction (a significant reduction in LVEF compared to the control group) as well as infarct size similarity in all animals before treatment, indicating no significant difference in initial ischemic injury between groups (Figure 1(c)). Histopathological analysis of the infarcted hearts revealed the presence of an infarcted zone with fibrosis and intense collagen deposition in the free wall of the left ventricle (see more details in Section 3.3).

### 3.2. Characterization of Expanded CD133^+^ Cells and Expanded CD133^+^ Cell-Derived EVs

After expansion, the phenotype of the CD133^+^ cell population acquired endothelial-like morphology observed under bright-field microscopy (Figure 1(d)). The cell viability was 90.31%, and the mean percentages of surface and intracellular markers were as follows: 18.8% CD133, 10.2% CD34, 0.5% CD45, 1.13% CD14, 100% CD105, 46% CD31, 60.4% CD309, 97.5% CD146, and 99.2% vWF (Figure 1(e)).

To evaluate the size distribution and concentration of EVs, the particles were examined using nanoparticle tracking analysis. The expanded CD133^+^ cell-derived EVs showed a heterogeneous population comprised of particles of 35 nm to 800 nm (Figure 1(f)). The total number of particles recovered from 2.2 × 10^8^ expanded CD133^+^ cells was 1.1 × 10^12^. On average, each expanded CD133^+^ cell produced 5.0 × 10^3^ EVs. Transmission electron microscopy revealed that most EVs were 30–100 nm in diameter, although larger and smaller vesicles were also observed (Figure 1(g)), consistent with the NTA results. EVs with spherical morphology and double membranes were also identified by TEM, as evidenced by the magnified images highlighted (Figure 1(g)). TEM associated with immunogold staining showed expanded CD133^+^ cell-derived EVs positive for CD31 and for the tetraspanin CD63, indicating the presence of microvesicles and exosomes in the purified EV sample (Figure 1(g)).

### 3.3. Effect of Expanded CD133^+^ Cells and Expanded CD133^+^ Cell-Derived EVs on Remodeling and the Cardiac Function of Infarcted Hearts

In the vehicle group, a mortality rate of 25% (2 of 8) was observed after 10 and 11 days of transplantation. Interestingly, no death was registered in the group that received expanded CD133^+^ cells or expanded CD133^+^ cell-derived EVs during the time course of this study, although no statistically significant differences were observed between the groups.

**Figure 2:**
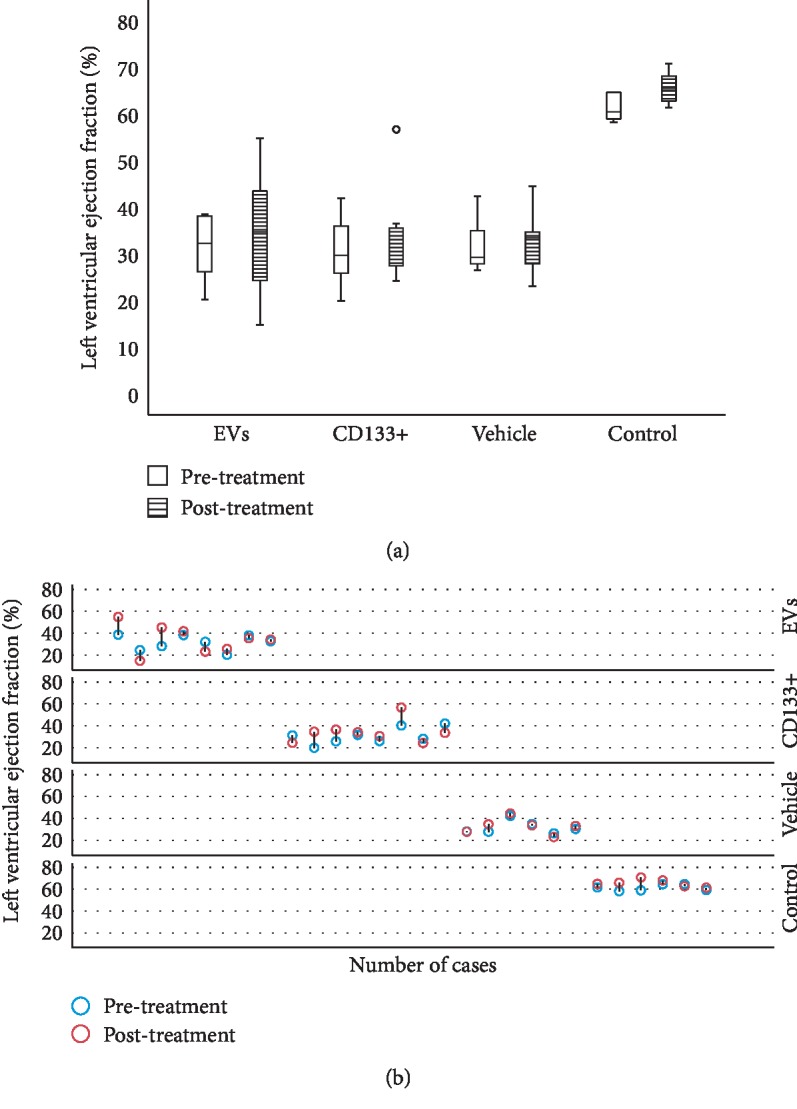
Echocardiographic evaluation of left ventricular ejection fraction. (a) Comparison of the left ventricular ejection fraction within each group before and after transplantation. (b) Left ventricular ejection fraction percentage of each animal pre- (blue circle) and post- (red circle) treatment. Data are presented as median and minimum and maximum values. °: outlier value; LVEF: left ventricular ejection fraction; EVs: transplanted with expanded CD133^+^ cell-derived EVs; CD133^+^: transplanted with expanded CD133^+^ cells; vehicle: treated with PBS; control: healthy animals (*n* = 6 to 8 per group).

Our previous work demonstrated that the intramyocardial transplantation of expanded CD133^+^ cells improved heart function in rats and is suitable for the regeneration of the vascular system [9]. To assess a more real and less invasive therapeutic approach, we tested the efficacy of the intravenous transplant of expanded CD133^+^ cells and EVs derived from CD133^+^ cell cultures in this well-established preclinical model of AMI. Echocardiographic analysis of cardiac function was carried out four weeks posttreatment. The results showed no statistically significant increase in LVEF pre- and posttreatment in the groups that received expanded CD133^+^ cells or expanded CD133^+^ cell-derived EVs. In addition, no significant differences were observed between the vehicle and treated groups before and after treatment (Figure 2). Although no statistically significant differences were observed in the global LVEF assessment, when each animal was analyzed individually, an expressive increase in LVEF was observed in some animals that received treatment (either expanded CD133^+^ cells or EVs, Figure 2(b)). In the group that received expanded CD133^+^ cell-derived EVs, two of eight animals showed an improved LVEF to a greater extent, from 38.6 to 54.8 (Δ = 16.2) and from 28.1 to 45.2 (Δ = 17.1), while in the group that received expanded CD133^+^ cells, three of eight animals showed an expressive increase in LVEF, from 19.9 to 34.6 (Δ = 14.7), from 25.8 to 36.5 (Δ = 10.7), and from 40.3 to 56.7 (Δ = 16.4). On the other hand, our results also showed that two animals from each treated group had an expressive decrease in LVEF ([Supplementary-material supplementary-material-1]). This behavior was not observed in the vehicle group, where the animals that survived did not present the same variations, either positive or negative, in LVEF.

To evaluate heart function and remodeling, the end-systolic and end-diastolic volumes of the left ventricle pre- and posttransplant were analyzed. The values of end-systolic and end-diastolic volumes significantly increased at 28 days posttransplant in all infarcted groups compared to the animals of the control group (Table 1).

**Figure 3:**
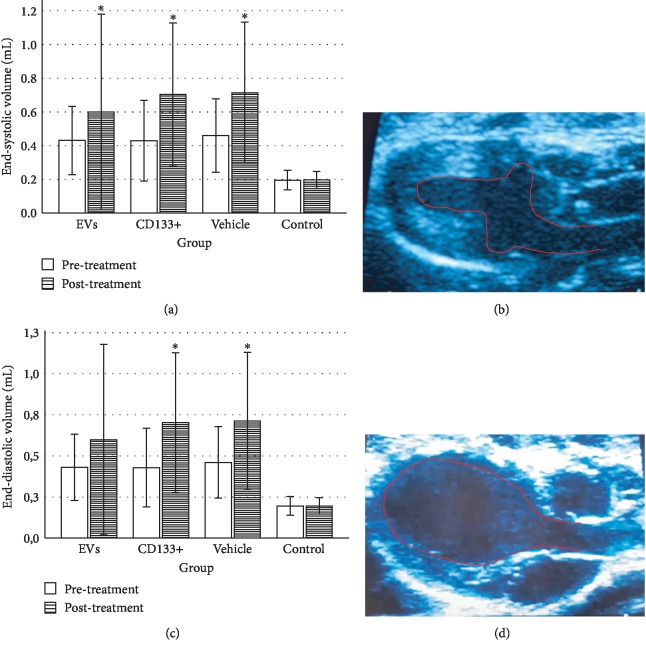
Echocardiographic evaluation of end-systolic and end-diastolic volume. Comparison of left ventricular end-systolic volume (a) and left ventricular end-diastolic volume (c) within each group pre- and posttreatment. Representative two-dimensional echocardiographic images of end-systolic volume (b) and end-diastolic volume (d) in a healthy animal of the control group. EVs: transplanted with expanded CD133^+^ cell-derived EVs; CD133^+^: transplanted with expanded CD133^+^ cells; vehicle: treated with PBS; control: healthy animals. ^∗^Significant difference between pre- and posttreatment moments of each group (*P* > 0.05). Data are represented as median, minimum and maximum values, and SEM bars (*n* = 6 to 8 per group).

No significant differences were observed in the ESV or EDV 28 days posttreatment among the vehicle and treated groups (Figures 3(a) and 3(b)). However, the group that received expanded CD133^+^ cell-derived EVs exhibited a small preservation of end-systolic and end-diastolic volumes ranging from 0.40 mL to 0.57 mL and from 0.60 mL to 0.85 mL, respectively, while in the vehicle group, these values ranged from 0.39 mL to 0.71 mL and from 0.67 to 1.0 mL, respectively. Although no statistically significant differences were observed in the global assessment of ESV and EDV among the infarcted groups, when each animal was analyzed individually, a slight decrease in ESV was observed in two animals that received expanded CD133^+^ cell-derived EVs. One animal showed a decrease in ESV from 0.367 mL to 0.259 mL (Δ = −0.108) and the other one from 0.443 mL to 0.36 mL (Δ = −0.083). Interestingly, the exact same two animals that showed a decrease in ESV were also the same that showed an expressive increase in LVEF ([Supplementary-material supplementary-material-1]). Although no decrease in the ESV was observed in the animals that received expanded CD133^+^ cells, two of three animals that demonstrated an expressive increase in LVEF maintained a similar ESV pre- and posttreatment.

**Figure 4:**
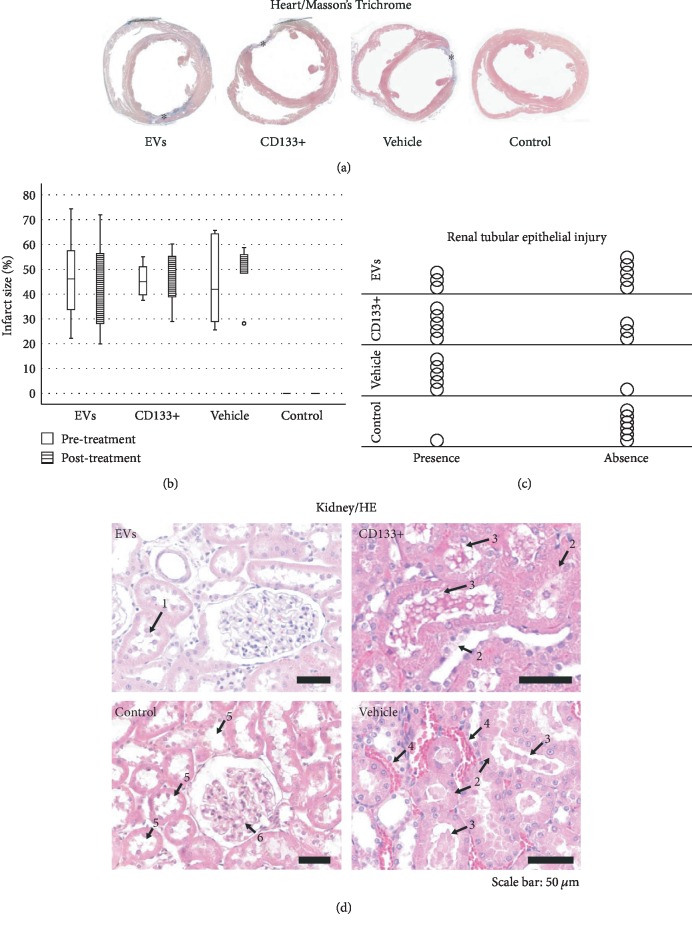
Effect of expanded CD133^+^ cells and expanded CD133^+^ cell-derived EVs on infarct size and renal injury. (a) Representative Masson's trichrome-stained section of hearts from each of the four groups. Blue staining is showing collagen deposition after myocardial infarction induction. (b) Echocardiographic evaluation of infarct size within each group before and after transplantation. (c) Comparison of the occurrence of renal tubular epithelial lesion between groups. Each circle in the graphic represents an animal. (d) Representative micrographs of renal histology from each of the four groups at day 28 after treatment administration. Arrow number 1 indicates evidence of renal necrotic cells flaking off to the tubular lumen, number 2 shows cell necrosis from the tubular wall, number 3 indicates evidence of renal tubules with Tamm-Horsfall (THP) protein, number 4 indicates vascular congestion, and number 5 indicates evidence of corpuscles while arrow number 6 shows preserved renal tubules in control healthy group. °: outlier value; EVs: transplanted with expanded CD133^+^cell-derived EVs; CD133^+^: transplanted with expanded CD133^+^ cells; vehicle: treated with PBS; control: healthy animals (*n* = 6 to 8 per group).

Reliable and reproducible assessment of the infarct size is vital in determining heart failure and death. In this study, we used echocardiography, an approach widely used in clinical analysis for the measurement of infarct size. Data from echocardiography revealed no statistically significant difference in the scar size pre- and posttreatment within and among the infarcted groups (Figure 4(b)). However, although no statistically significant differences were observed in the global assessment of infarct size, two animals treated with expanded CD133^+^ cell-derived EVs showed an expressive decrease in the infarct size, from 35.16 to 19.95 (Δ = −15.21) and from 47.6 to 25.87 (Δ = −21.73) ([Supplementary-material supplementary-material-1] and Figure 4(a)). It is worth noting that these two animals also exhibited a substantial increase in LVEF as well as a decrease in the ESV posttreatment. Interestingly, one of three animals that exhibited an expressive increase in LVEF after treatment with expanded CD133^+^ cells also presented a decrease in the infarct size, from 45 to 40 (Δ = −5).

### 3.4. In Vivo Effects of Expanded CD133^+^ Cells and Expanded CD133^+^ Cell-Derived EVs on Renal Injury in Infarcted Rats

In addition to the infarct size, we looked for evidence of renal injury. Several studies have shown that congestive heart failure is a major cause of progressive chronic kidney injury and, conversely, that chronic kidney disease itself is a major contributor to severe cardiac damage. In this study, histological analyses were carried out on the left and right kidneys 28 days after treatment. In the vehicle group, 83.3% (5 of 6) of the animals showed signs of renal injury, as evidenced by the formation of vascular congestion, necrotic cells on renal tubules, and presence of renal tubules with Tamm-Horsfall (THP) protein (Figures 4(c) and 4(d)). Interestingly, less morphological injuries were observed in the group that received expanded CD133^+^ cells (62.5%) and expanded CD133^+^ cell-derived EVs (37.5%) resembling the control group (Figures 4(c) and 4(d)). It is worth noting that the animals that exhibited an expressive increase in LVEF after treatment with expanded CD133^+^ cells or expanded CD133^+^ cell-derived EVs were also the same animals that did not exhibit renal injury.

## 4. Discussion

We have previously profiled the protein content of EVs derived from human expanded CD133^+^ cells. Gene Ontology analyses revealed that the proteins are involved in a variety of angiogenesis-related functionalities and, also, in the proper functioning of the renal filtration barrier, with both structural and signaling functions [18]. In order to test the presumed functions of the expanded CD133^+^ cells and their EVs, we conducted a preclinical study where we evaluated the effectiveness of the systemic application of the cells and their EVs for the treatment of ischemic cardiomyopathy in a rat model of AMI.

We demonstrated that the protocol for the induction of acute myocardial infarction in rats was effective and showed similarity to the morphological and functional changes that occur after myocardial infarction in humans, as previously described [23]. The AMI group exhibited a significant decrease in LVEF and a substantial increase in the ESV, EDV, and infarct size. In addition, histopathological analyses revealed that infarcted hearts had histopathological characteristics that were similar to those that have been reported by others [24, 25]. To the best of our knowledge, no previous study has evaluated the effect of the systemic application of expanded CD133^+^ cells and EVs derived from the same cell source for the treatment of ischemic cardiomyopathy. The echocardiographic results showed no significant differences in the recovery of left ventricular function among the vehicle and treated groups after transplantation. In addition, data from echocardiography revealed no significant differences in the scar size pre- and posttreatment within and among the infarcted groups.

Although no significant difference was observed in the global assessment of heart function and remodeling among the infarcted groups, it is worth highlighting that some animals treated with either expanded CD133^+^ cells or expanded CD133^+^ cell-derived EVs exhibited a consistent positive correlation between all cardiac and renal parameters analyzed. Three of eight animals treated with expanded CD133^+^ cells that showed a huge increase in LVEF also showed an absence of renal injury, and one animal had a decrease in the infarct size. Interestingly, two of eight animals treated with expanded CD133^+^ cell-derived EVs showed an expressive increase in LVEF along with a reduction in ESV and infarct size and the absence of renal injury. Expanded CD133^+^ cells or expanded CD133^+^ cell-derived EVs seem to have the potential to improve cardiac function and reduce renal damage; however, it seems that dose adjustments and/or alternative routes need to be tested. Although all the animals had an LVEF < 45% before transplantation, the infarct model used in this study led to a wide variation in ejection fraction, ranging from approximately 20% to 45% in all groups.

Several preclinical studies that have used the injection of endothelial progenitor cells [5, 26, 27] or expanded CD133^+^ cells [7, 28] directly into a damaged heart have shown improved global heart function and decreased cardiac fibrosis. Khan and coworkers [29] demonstrated that mouse ESC-derived exosomes injected directly into the MI border zone have the ability to enhance neovascularization and cardiomyocyte survival and reduce fibrosis postinfarction. In addition, previous studies have demonstrated that the intramyocardial administration of EVs results in heart function improvement in infarcted hearts [30, 31]. Although the direct intracardiac injection of stem/progenitor cells or EVs has been used in animal studies in an attempt to optimize heart function after myocardial injury, this technique has been considered a more invasive practice that could lead to increased animal death. In this regard, other routes of administration still need to be explored and tested. In our study, we investigated a systemic administration protocol that could be easily applied in clinical studies. We demonstrated that the systemic application of expanded CD133^+^ cells or expanded CD133^+^ cell-derived EVs, in fact, was not an appropriate route for the treatment of ischemic cardiomyopathy.

Previous studies have shown that after systemic application, EVs tend to accumulate mainly in the liver, followed by the spleen, gastrointestinal tract, and lungs [32, 33]. In addition, one of the research groups also reported differences in EV localization when they were administered subcutaneously, intraperitoneally, or intravenously, showing that different routes of systemic application could influence EV tissue distribution. We hypothesized that EVs administered by a systemic route may be retained in other organs, with poor accumulation in the heart. Grange and coworkers [32] reported that labeled EVs displayed a greater specificity for the injured kidney compared to healthy ones, demonstrating the ability of EVs to localize with renal disease. Several studies have shown evidence that a derangement of cardiac function may induce acute or chronic kidney injury [3, 34]. In our study, we observed that more than 65% of the animals treated with expanded CD133^+^ cell-derived EVs did not exhibit renal injury. The specificity of EVs to accumulate in injured kidneys along with their poor distribution in the heart may explain why in our study, we did not observe a substantial beneficial effect of systemic application on heart function recovery following myocardial infarction. Moreover, in this study, 50 *μ*g of EVs were administered by systemic application. We speculate that achieving a beneficial effect on the heart by a systemic route might require a higher dose of vesicles or even multiple applications.

## 5. Conclusion

In conclusion, we demonstrated that the systemic application of expanded CD133^+^ cells and EVs has similar effects in infarcted rats. Few animals per group showed improvements in several heart and kidney parameters analyzed, but not significant differences were observed when comparing the groups. The systemic route may not be effective to treat ischemic cardiomyopathy; nonetheless, it may be a beneficial therapy to treat the side effects of AMI such as kidney damage.

## Figures and Tables

**Table 1:** Echocardiographic parameters of the left ventricle pre- and posttreatment.

Group	LVEF (%) Pretreatment	LVEF (%) Posttreatment	EDV (mL) Pretreatment	EDV (mL) Posttreatment	ESV (mL) Pretreatment	ESV (mL) Posttreatment
EVs	29.7 (19.9/42)	33.8 (24.3/56.7)	0.60 (0.45/0.76)	1.02 (0.71/1.35)	0.41 (0.27/0.60)	0.72^∗^ (0.31/1.02)
CD133+	32.3 (20.2/38.6)	34.7 (14.8/54.8)	0.62 (0.44/0.75)	0.88^∗^ (0.54/1.32)	0.45 (0.27/0.56)	0.59^∗^ (0.25/1.12)
Vehicle	29.3 (26.5/42.4)	33.5 (23.1/44.5)	0.67 (0.44/0.84)	1.12^∗^ (0.67/1.36)	0.46 (0.31/0.60)	0.68^∗^ (0.44/0.98)
Control	60.4 (58.3/64.7)	65.3 (61.4/70.8)	0.49 (0.42/0.60)	0.57 (0.52/0.61)	0.19 (0.16/0.24)	0.18 (0.17/0.23)

LVEF: left ventricular ejection fraction; EDV: end-diastolic volume; ESV: end-systolic volume; EVs: transplanted with expanded CD133^+^ cell-derived EVs; CD133^+^: transplanted with expanded CD133^+^ cells; vehicle: treated with PBS; control: healthy animals (*n* = 6 to 8 per group). Data are presented as median (minimum/maximum). ^∗^Significant difference between pre- and posttreatment moments of each group (*P* < 0.05).

## Data Availability

The data used to support the findings of this study are available from the corresponding authors upon request.

## Supplementary Materials

Supplementary Materials Supplemental information includes one Excel file with all the measurements obtained for LVEF, EDV, ESV, and infarct size pre- and posttreatment which can be found with this article online.
